# Intermediate host patterns of acanthocephalans in the Weser river system: co-invasion *vs* host capture

**DOI:** 10.1017/S0031182023000124

**Published:** 2023-04

**Authors:** Sebastian Vogel, Horst Taraschewski

**Affiliations:** 1 Karlsruhe Institute of Technology (KIT), Zoological Institute, Karlsruhe, Germany; 2 Department of Palaeontology and Evolution, Staatliches Museum für Naturkunde Karlsruhe (SMNK), Karlsruhe, Germany

**Keywords:** Ecological globalization, host specificity, invasive species, *Paratenuisentis*, *Polymorphus*, *Pomphorhynchus*, taxonomic DNA barcoding, xenodiversity

## Abstract

Anthropogenic interference is a major driver of ecological change in freshwater ecosystems. Pollution and the introduction of new species not only alter macrozoobenthic community structures, but can also affect their respective parasite communities. The ecology of the Weser river system experienced a drastic decline in biodiversity over the past century due to salinization caused by the local potash industry. As a response, the amphipod *Gammarus tigrinus* was released into the Werra in 1957. A few decades after the introduction and subsequent spread of this North American species, its natural acanthocephalan *Paratenuisentis ambiguus* was recorded in the Weser in 1988, where it had captured the European eel *Anguilla anguilla* as a novel host. To assess the recent ecological changes in the acanthocephalan parasite community, we investigated gammarids and eel in the Weser river system. In addition to *P. ambiguus*, 3 *Pomphorhynchus* species and *Polymorphus* cf. *minutus* were discovered. The introduced *G. tigrinus* serves as a novel intermediate host for the acanthocephalans *Pomphorhynchus tereticollis* and *P.* cf. *minutus* in the tributary Werra. *Pomphorhynchus laevis* is persistent in the tributary Fulda in its indigenous host *Gammarus pulex*. *Pomphorhynchus bosniacus* colonized the Weser with its Ponto-Caspian intermediate host *Dikerogammarus villosus*. This study highlights the anthropogenically driven changes in ecology and evolution in the Weser river system. Based on morphological and phylogenetic identification, the shifts in distribution and host usage described here for the first time contribute to the puzzling taxonomy of the genus *Pomphorhynchus* in times of ecological globalization.

## Graphical abstract

**Figure:**
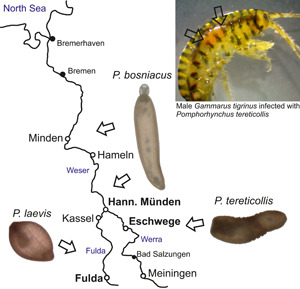

## Introduction

The German river Weser along with its tributary Werra has a long history of salt pollution. Since the early 20th century, discharge of waste waters by the local potash mining industry into the Werra severely increased the river's salinity (Braukmann and Böhme, 2011; Schulz and Cañedo-Argüelles, 2019). Subsequently, the decrease in water quality led to an impoverished diversity of macrozoobenthic organisms (Hübner, 2007). To replace the disappeared indigenous *Gammarus* species, the euryhaline *Gammarus tigrinus* Sexton, 1939 was intentionally released into the Werra by Schmitz in 1957. This North American amphipod species quickly established a dense population, spreading beyond the Weser–Werra–Estuary into other river systems and the Baltic Sea (Bulnheim, 1976; Rewicz *et al*., 2019; Spikkeland *et al*., 2020).

The first infections of *G. tigrinus* with the acanthocephalan *Paratenuisentis ambiguus* (Van Cleave, 1921) in the Weser were discovered a few decades after the initial release of the alien amphipod (Taraschewski *et al*., 1987). In its recipient range as well as its native area, *P. ambiguus* has been recorded from *G. tigrinus* as well as the indigenous eel species *Anguilla anguilla* (Linnaeus, 1758) and *Anguilla rostrata* (Lesuer, 1817) as final host (Gilbert and Bullock, 1981; Taraschewski *et al*., 1987).

In comparison, species of *Pomphorhynchus* have been recorded from a variety of intermediate and definitive hosts. However, the data available are masked by the puzzling taxonomy of this acanthocephalan genus in Europe. Nowadays, the *Pomphorhynchus* species complex in Central Europe is considered to comprise at least 3 species, *Pomphorhynchus tereticollis* (Rudolphi, 1809), *Pomphorhynchus laevis* (Zoega in Müller, 1776) and *Pomphorhynchus bosniacus* Kiskároly & Čanković, 1969. These congeners can be well distinguished on a molecular level but express a somewhat similar polymorphic morphology that may have led to misidentifications in the past (Špakulová *et al*., 2011; Perrot-Minnot *et al*., 2018; Reier *et al*., 2019).

After a long period of taxonomic dis- and reassembling, *P. tereticollis* was conclusively reestablished as distinguishable from *P. laevis* (Špakulová *et al*., 2011). Several studies focused on the replacement of these *Pomphorhynchus* species by each other, following the recent range expansion of their respective intermediate and paratenic hosts (Emde *et al*., 2012; David *et al*., 2018; Hohenadler *et al*., 2018). However, no differentiation between *P. bosniacus* and *P. laevis* was made in these studies, as the distinctiveness of both species had not yet been revealed (Reier *et al*., 2019). Furthermore, information on the host–parasite relationship of previous invaders, such as *G. tigrinus*, became scarce, as most authors focused on the Ponto-Caspian invasion wave of amphipods, especially *Dikerogammarus villosus* (Sowinsky, 1894) (Daunys and Zettler, 2006; Kornis *et al*., 2012; Reisalu *et al*., 2016; MacNeil, 2019). Until now, *G. tigrinus* had not been reported to serve as an intermediate host of a *Pomphorhynchus* species in Europe. In its native area of North America, *G. tigrinus* is parasitized by *Pomphorhynchus rocci* Cordonnier & Ward, 1967 (Johnson and Harkema, 1971). However, thus far *P. rocci* failed to colonize European populations of *G. tigrinus*.

In addition to the species debate within the *Pomphorhynchus* genus, the taxonomic resolution of other acanthocephalans, such as *Polymorphus minutus* (Goeze, 1782), is equally intricate. This parasite of waterbirds may share some intermediate hosts, such as *Gammarus pulex* (Linnaeus, 1758), with *Pomphorhynchus* spp. (Kaldonski *et al*., 2008). However, recent advances in phylogenetic research led to the emergence of cryptic species of *P.* cf. *minutus* that differ greatly in their intermediate host specificity (Zittel *et al*., 2018; Grabner *et al*., 2020).

Since the intentional release of *G. tigrinus* in the Weser river system, the arrival of other alien species such as *D. villosus* increased the local xenodiversity, as these non-indigenous species now account for the largest proportion of the macrozoobenthic community composition (Grabow *et al*., 1998). After the initial studies on host–parasite relationships by Taraschewski *et al*. (1987) little to no efforts have been made to evaluate the recent parasitical developments in this system.

To address the informational gap, we investigated gammarids and their acanthocephalan parasites in the river Werra. After the discovery of *P. tereticollis* in *G. tigrinus*, the main objective of this study was to compare the intermediate host patterns of acanthocephalans in the Weser river system. Regarding the challenging taxonomy of *Pomphorhynchus* a combination of biological, morphological and phylogenetic means of differentiation was chosen. For non-indigenous acanthocephalans, whether their range extension was driven by host–parasite co-invasion or by host capture is discussed.

## Materials and methods

### Sampling

**Fig. 1.**
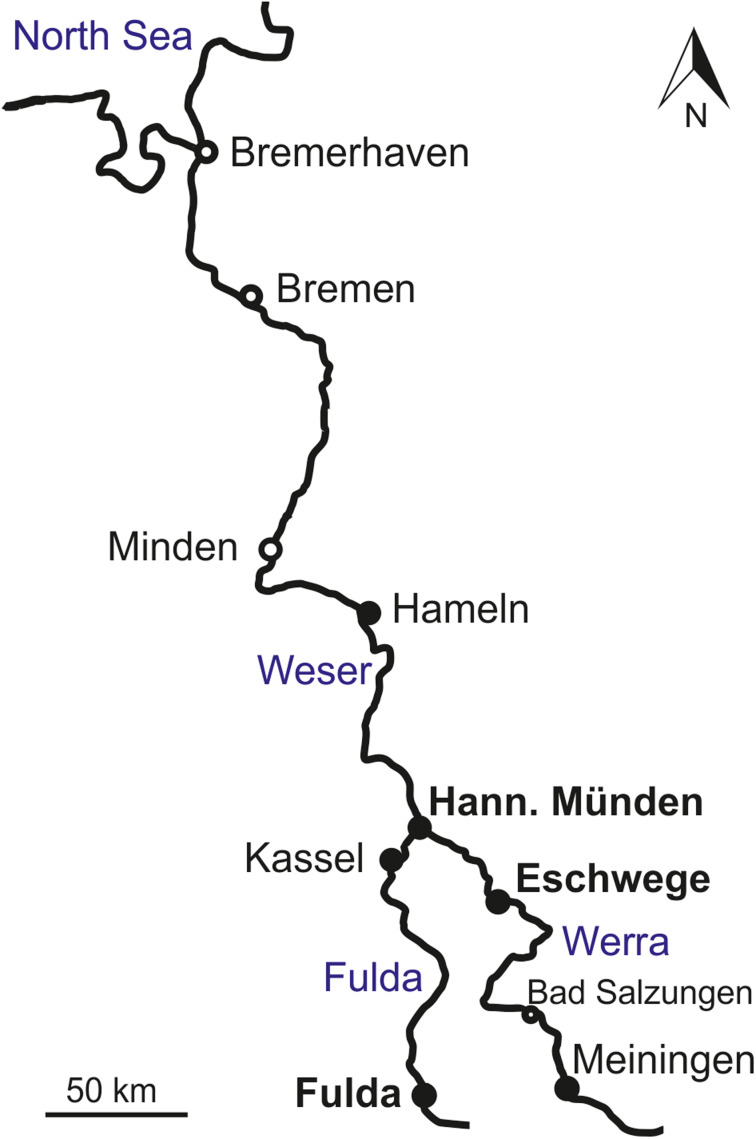
Location of the study sites along the Weser river system in northern Germany (full circles).

Six sites were chosen for gammarid acquisition between 2018 and 2021 (Fig. 1). The sites consist of the Werra in Eschwege (51°11′28.2″N, 10°03′24.9″E) and Meiningen (50°34′04.5″N, 10°24′33.2″E), the Fulda in the cities Fulda (50°33′16.3″N, 9°39′52.0″E) and Kassel (51°17′20.2″N, 9°29′25.2″E) and the Weser near Hannoversch Münden (51°28′07.7″N, 9°38′42.1″E) and Hameln (52°06′32.0″N, 9°20′59.3″E). Amphipods were captured *via* kick-sampling using a hand sieve (2 mm mesh width), transported to the laboratory alive and deep-frozen until dissection. The sex, body length from head to urosome excluding the uropod, length of the second antennae as well as the infection status were recorded for each gammarid. In most cases, infected specimens could be easily recognized by the orange-red colour of the cystacanth (Fig. S1a). Prevalence [*P* (%)] as well as mean intensity of infection (MI) were calculated for each sample (Table 1). To enhance passive proboscis evagination, cystacanths from defrost amphipods were maintained overnight in distilled water at 8°C. Photographs of whole, unfixed specimens were taken using a Kayence VHX-7000 digital microscope. The samples were then preserved in 95% ethanol for molecular analysis.

**Table 1.** Amphipod populations infected with *Pomphorhynchus* spp., according to sampling month and number of (infected) hosts per sample.

Host	05/2018	03/2019	09/2019	03/2020	07/2020	09/2020	06/2021
*Gammarus tigrinus*^1^	192 _(26)_	142 _(11)_	238 _(19)_	584 _(71)_	2240 _(10)_	159 _(1)_	774
*P* (%) _(MI)_	13.5 _(1.2)_	7.7 _(1)_	8 _(1.1)_	12.2 _(1.1)_	0.4 _(1)_	0.6 _(1)_	–
*Dikerogammarus villosus*^2^	97 _(2)_	–	173	156	262 _(2)_	–	178
*P* (%) _(MI)_	2.1 _(1)_	–	–	–	0.8 _(1)_	–	–
*Gammarus pulex*^3^	–	–	153 _(1)_	138 _(8)_	494 _(4)_	85 _(6)_	491
*P* (%) _(MI)_	–	–	0.7 _(2)_	5.8 _(1.4)_	0.8 _(1)_	7.1 _(1.2)_	–

*P* (%): prevalence; MI: mean intensity of infection.

1 Eschwege.

2 Hann. Münden.

3 Fulda.

In addition, adult specimens of *P. bosniacus* were obtained by dissecting the intestines of frozen eels gathered from local fishermen in Nienburg and Landesbergen. We were assured that all fish originated from the river Weser. Specimens obtained from the dissections were stored in 95% ethanol until molecular analysis.

### Morphological identification

*Paratenuisentis ambiguus* (Fig. S1b) was identified based on its original description and subsequent records from the Weser river system (Bullock and Samuel, 1975; Taraschewski *et al*., 1987). *Polymorphus* cf. *minutus* was recognizable by its bright colour and surrounding envelope (Fig. S1c) (Dezfuli and Giari, 1999). A single specimen was used for DNA barcoding according to Zittel *et al*. (2018). The morphological identification of *Pomphorhynchus* spp. (Fig. S1d–f) was performed using the keys available (Amin *et al*., 2003), as well as the criteria described by Špakulová *et al*. (2011) and the observations from Reier *et al*. (2019).

### DNA barcoding

**Fig. 2.**
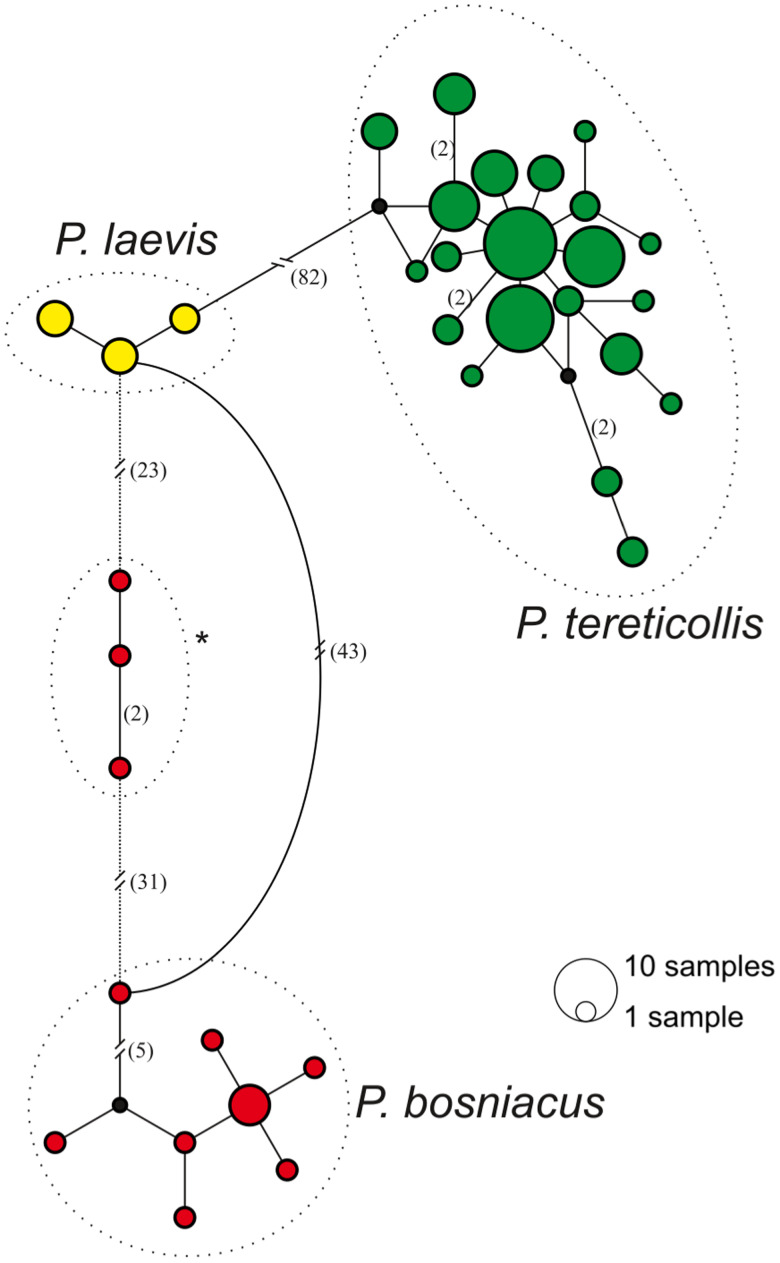
MJ haplotype network constructed with PopART containing 95 sequences obtained in this study. * indicate possible pseudogenes.

To ensure the correct taxonomic placement of *Pomphorhynchus* specimen, a DNA barcoding approach was chosen. DNA was extracted using Chelex^®^ and amplification of cytochrome c oxidase subunit I (COI) was performed according to Tierney *et al*. (2020). Polymerase chain reaction products were sent to Microsynth Seqlab for Sanger sequencing using the forward primer. Ninety-five sequences of 507 bp length were obtained: 73 *P. tereticollis* from *G. tigrinus*; 12 *P. bosniacus* from *A. anguilla*, 2 from *D. villosus* and 8 *P. laevis* from *G. pulex*. Initial alignment was performed using ClustalW in MEGA 11 (Kumar *et al*., 2018), before constructing median-joining (MJ) haplotype networks (Bandelt *et al*., 1999) in PopART (https://popart.maths.otago.ac.nz/) (Leigh and Bryant, 2015) (Fig. 2).

**Fig. 3.**
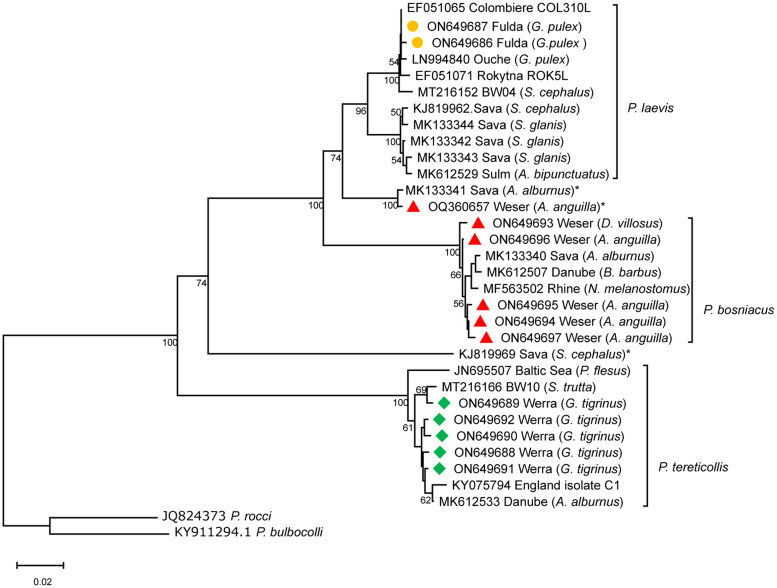
NJ phylogenetic tree computed using MEGA 11. Numbers indicate branch support assessed by 1000 bootstrap iterations (values below 50 are not shown). Titles consist of NCBI accession number, sampling location and host (if available). Coloured markers indicate sequences obtained in this study. * indicate potential pseudogenes or mtDNA-like sequences.

For further phylogenetic analysis, the following NCBI accessions were added to the dataset EF051062–EF051071 (Moret *et al*., 2007), JN695505–JN695508 (Špakulová *et al*., 2011), JQ824373 (Pan and Nie, 2012, unpublished), KJ819957–KJ820006 (Vardić Smrzlić *et al*., 2015), KY075794 (Andreou *et al*., 2020), KY490045–KY490047 (Li *et al*., 2017), KY911293–KY911323 (García-Varela *et al*., 2017), LN994840–LN994853 (Perrot-Minnot *et al*., 2018), MF563495–MF563527 (David *et al*., 2018), MK133340–MK133344 (Nedić *et al*., 2019) and MK612497–MK612545 (Reier *et al*., 2019), MT216151–MT216172 (Ros *et al*., 2020) (for more details see the Supplementary material). The total dataset of 316 sequences was edited in MEGA 11 and trimmed to a length of 507 bp. An MJ haplotype network was constructed for each *Pomphorhynchus* lineage (Fig. S2a–c). We chose a subset of 33 sequences to construct a neighbour-joining (NJ) tree in MEGA 11 using uncorrected *P*-distances (Fig. 3). Branch support was assessed with 1000 bootstrap replicates and partial deletion was chosen as missing data treatment to accommodate for sequences shorter than 507 bp.

## Results

### Amphipod distribution

For each of the sampled river sections, a distinct community composition of Amphipoda was observed (Table 2). In the Werra section impacted by high salinity, *G. tigrinus* was the main component of the macrozoobenthic community (*n* = 4329). Upstream of the salt discharge sites in the Werra, only a small abundance of gammarids was encountered. The few amphipods obtained (*n* = 22) belonged to *Gammarus roeselii* (Gervais, 1835).

**Table 2.** Distribution of amphipods and acanthocephalans in the investigated area

Sample location	Host	*N*	*N*_inf_	Acanthocephalan	*P* (%)	MI
Eschwege^1^	*G. tigrinus*	4329	7	*Paratenuisentis ambiguus*	0.2	1
			138	*Pomphorhynchus tereticollis*	3.2	1.1
			2	*Polymorphus minutus*	0.05	1
	*Apocorophium lacustre*	7	–			
Meiningen^1^	*Gammarus roeselii*	22	–			
Hameln^2^	*D. villosus*	167	–			
	*G. tigrinus*	2	–			
Hann. Münden^2^	*D. villosus*	866	4	*Pomphorhynchus bosniacus*	0.5	1
	*G. pulex*	1	–			
	*G. roeselii*	1	–			
	*Chelicorophium curvispinum*	2	–			
Kassel^3^	*D. villosus*	1300	–			
Fulda^3^	*G. pulex*	1363	19	*Pomphorhynchus laevis*	1.4	1.3
			1	*P. minutus*	0.07	2
	*G. roeselii*	45	–			

*N*: total number of amphipods; *N*_inf_: number of infected hosts; *P* (%): prevalence; MI: mean intensity of infection.

1 Werra.

2 Weser.

3 Fulda.

The Weser at Hannoversch Münden was populated by a quantity of *D. villosus* (*n* = 866), in addition to single records of *Chelicorophium curvispinum* (Sars, 1895), *G. pulex* and *G. roeselii*. Similarly, the sampling site in Hameln was dominated by *D. villosus* (*n* = 167). Here, 2 specimens of *G. tigrinus* were recorded outside the river Werra. The lower Fulda at Kassel was not distinct from the Weser samples, as only *D. villosus* was found here (*n* = 1300). More upstream, the river Fulda at the city Fulda was inhabited by *G. pulex* (*n* = 1361) and *G. roeselii* (*n* = 45).

### Parasite distribution

*Paratenuisentis ambiguus* was found in low prevalence (0.2 ± 0.5%) in *G. tigrinus* from the Werra throughout the sampling period and only a small quantity of adults (*n* = 3) could be obtained from the required definitive host *A. anguilla* in the Weser in 2018. General prevalence of *P. tereticollis* in *G. tigrinus* in the river Werra was different when comparing total [*P* (%): 3.2] and mean prevalence (6.1 ± 6.6%), but specimens were found throughout the investigation period (Table 1). During sampling in 2019, 2 cystacanths of waterbird-parasitizing *P.* cf. *minutus* were also discovered in *G. tigrinus*. However, prevalence in the single sample was low (0.2%) and no further infections could be recorded.

Four specimens of *D. villosus* harboured *P. bosniacus*, although prevalence in the intermediate host was generally low [*P* (%): 0.5]. However, the intestinal parasite community of the eel investigated (*n* = 48) was dominated by subadult *P. bosniacus* [*P* (%): 72.9; MI: 5.7], of which only 19.9% truly penetrated the intestinal wall while the rest were merely attached to it. No gravid females were discovered.

*Pomphorhynchus laevis* was collected from *G. pulex* in small abundance during samplings of the Fulda in 2020, where prevalence ranged between 0.7 and 5.8, with a total prevalence of 1.4.

### Morphological identification

Cystacanths of *Pomphorhynchus* spp. showed a different shape (Fig. S1d–f) and proboscis hook formula depending on river section of origin and intermediate host, respectively. *Pomphorhynchus tereticollis* had an elongated, cylindrical trunk with a wrinkled surface in most, though not all larvae (Fig. S1d), and a proboscis armed with 14–20 longitudinal rows of 9–12 hooks each. Length of the relaxed cystacanths ranged between 2.9 and 4.9 mm. The few specimens obtained from the Weser section near Hannoversch Münden (Fig. S1e) were of similar shape and length (3.5 mm) but had a smoother trunk and fewer hooks (7–9) in 14 longitudinal rows, therefore they were identified as *P. bosniacus*. In comparison, *P. laevis* larvae from the river Fulda (Fig. S1f) were of a more compressed, ovoid or spindle-like shape, smaller overall trunk size (1.0–2.9 mm) and armed with 16–20 rows of 10–13 hooks each.

### Phylogenetic analysis

A total of 83 cystacanths (2 *P. bosniacus*, 8 *P. laevis*, 73 *P. tereticollis*) and 12 subadult specimens (*P. bosniacus*) were used for phylogenetic analysis. All sequences obtained in this study clustered according to sampling location and host, thus confirming our morphological species identification. Four haplogroups could be differentiated in our dataset (Fig. 2), each of the 3 *Pomphorhynchus* species being represented by 1, in addition to a 4th haplogroup in between the main clusters of *P. bosniacus* and *P. laevis*. Mean genetic *P*-distance between this haplogroup and *P. bosniacus* is 0.084 ± 0.013, and 0.073 ± 0.01 between it and *P. laevis*, respectively. Distance between *P. bosniacus* and *P. laevis* in our samples is 0.112 ± 0.013. In the NJ tree (Fig. 3), the 3 sequences in this additional cluster appear closer to *P. laevis*, however, their placement is not supported.

## Discussion

### Amphipod distribution

Sampling the main streams of the Weser river system provided a characteristic gammarid community for each Werra, Fulda and Weser. *Gammarus tigrinus* was the main macrozoobenthic component in the Werra, and seems to be the only amphipod, besides *Apocorophium lacustre* (Vanhöffen, 1911), to inhabit the polluted river sections (Szöcs *et al*., 2014). Our results suggest *G. tigrinus* almost completely disappeared from the Weser, although it reached high abundances in the northern central Europe as well as the Baltic Sea following its introduction to Europe (Spikkeland *et al*., 2020). It was replaced by *D. villosus* that had been reported from the investigation area more than 20 years ago (Grabow *et al*., 1998). This Ponto-Caspian super-spreader, along other newly introduced amphipods, extended its range into central Europe following the inauguration of the Rhine–Main–Danube Canal (Grabow *et al*., 1998; Leuven *et al*., 2009; Rewicz *et al*., 2014). Considering its invasive potential, it has likely outcompeted *G. tigrinus* in German rivers, including the Weser. However, data from the Netherlands show that *G. tigrinus* can compete with the highly competitive *D. villosus* by retreating to deeper regions (Platvoet *et al*., 2009). Only shallow shores are accessible for kick-sampling, therefore, our results regarding the distribution of *G. tigrinus* in the Weser may be prone to sampling bias.

Although *D. villosus* successfully extended its range across the Weser into the upper Fulda, it has not colonized the Werra. Therefore, the fusion of Fulda and Werra forming the Weser at Hannoversch Münden likely acts as a salinity barrier for *D. villosus*, preventing its spread (Boets *et al*., 2010; Gallardo *et al*., 2012). With the emergence of the new xenodiversity in the Weser river system, native gammarids have been largely replaced. In the upper Fulda, *G. pulex* can still be found, although it does not seem to inhabit the river Weser anymore. The species has also been reported from unpolluted parts of the Werra (Braukmann and Böhme, 2011).

### Paratenuisentis ambiguus

Host–parasite interactions in the river Weser and its tributary Werra appear to be highly influenced by ecological globalization, as revealed by the 5 acanthocephalan species present in this study. About 35 years ago, the successful jump invasion of the American eel-specific acanthocephalan *P. ambiguus* was discovered, when it adopted the European eel as a novel final host and retained the American amphipod *G. tigrinus* as its intermediate host (Taraschewski *et al*., 1987). *Gammarus tigrinus* was intentionally released into the Werra in 1957, after the indigenous gammarid fauna disappeared due to salinization by potash mining effluents (Schmitz, 1960; Bulnheim, 1976). The introduced specimens were raised in a laboratory; therefore, it is unlikely that *P. ambiguus* invaded the habitat with its intermediate host. Supporting this assumption, there usually exists a lag of one to several decades between colonization by a novel host and the arrival of its parasite (Taraschewski, 2006).

As shown in this study, the natural acanthocephalan parasite of *G. tigrinus*, *P. ambiguus*, is still reminiscent in the Weser river system, although drastically declined in comparison to the previously reported high abundance (Taraschewski *et al*., 1987). *Paratenuisentis ambiguus* has been reported from *G. tigrinus* in the river Rhine, coastal waters of Poland and the northern Vistula Lagoon in Russia (Sures *et al*., 1999; Rodjuk and Shelenkova, 2006; Dzido *et al*., 2020), but to our best knowledge there are no other records of this acanthocephalan in Europe. A contributing factor to the narrow ecological amplitude of this brackish water-transmitted acanthocephalan might be its dependence on eels and on *G. tigrinus*. The jump invasion into Europe coincided with the host capture of *A. anguilla*, but thus far no intermediate host other than *G. tigrinus* has been reported to be infected with *P. ambiguus*, neither in North America nor in the novel area Europe.

### Pomphorhynchus

Other genera of Acanthocephala reveal a much more complicated taxonomy related to their invasion ecology. For several decades, *P. tereticollis* was considered synonymous with *P. laevis* until becoming reestablished based on morphological as well as molecular data (Špakulová *et al*., 2011). In contrast, the original description of *P. bosniacus* did not gain much response in literature and until its recent revival based on a combined morphological and genetic approach, this species was potentially misidentified as *P. laevis* in Central Europe (Kiskároly and Čanković, 1967; Reier *et al*., 2019).

Two lineages of *Pomphorhynchus*, herein referred to as *P. bosniacus* and *P. laevis* were identified in the Weser river system. The results presented in this study demonstrate that those 2 lineages differ greatly in their utilization of intermediate hosts in the Weser system, with *P. laevis* being found in *G. pulex* in the Fulda and *P. bosniacus* in *D. villosus* in the Weser, respectively. The distinction in the life cycle of both these acanthocephalans further affirms their consideration as 2 different species.

Additionally, the anatomical features of the Fulda and Weser specimen indicate a simple morphological distinction based on the formula of the proboscis hook armature might be possible, as *P. bosniacus* possessed fewer hooks per row than its congeners. On the contrary, cystacanths of *P. laevis* were of smaller size and more ovoid to spindle-like shape when compared to *P. bosniacus* and *P. tereticollis*. Differences in the morphology of cystacanths within the *Pomphorhynchus* species complex have been described previously (Perrot-Minnot, 2004), but were never utilized to a broader application as an additional criterion for species identification.

Inconsistencies in terms of genetic relationships and therefore taxonomy within the genus *Pomphorhynchus* based on the COI locus have been reported in various studies (Perrot-Minnot *et al*., 2018; Nedić *et al*., 2019; Mauer *et al*., 2020). Phylogenetic comparison of specimen from the Weser river system supports the existence of 3 separate species. *Pomphorhynchus tereticollis* in our sample was closely related to specimens from southern Germany and the British Isles (Andreou *et al*., 2020; Ros *et al*., 2020). Haplotype analysis and the NJ tree revealed that *P. laevis* belonged to the western-European clade previously described by Perrot-Minnot *et al*. (2018). Most COI sequences of *P. bosniacus* fitted well within the invasive lineage that has extended its range across the rivers Danube and Rhine in the last decade (David *et al*., 2018; Reier *et al*., 2019). However, 3 specimens clustered with a single sequence (MK133341) from the river Sava (Nedić *et al*., 2019). Based on host and distribution range, as well as morphological features, it was not possible to distinguish between these 2 *P. bosniacus* haplogroups. Given several base substitutions when compared to other *P. bosniacus* and *P. laevis* in our dataset, it is likely that these sequences represent mtDNA-like sequences or pseudogenes (Vardić Smrzlić *et al*., 2015; Nedić *et al*., 2019).

### Co-invasion

Although infection rate in general is low, *D. villosus* serves as an intermediate host of *P. bosniacus* in the colonized river Weser in Germany. This is further highlighted by the abundance of subadults found in the eels investigated from Landesbergen and Nienburg in 2018. The parasite seems to have followed the trail of its highly expansive Ponto-Caspian amphipod, colonizing river systems in central, northern and western Europe (Rewicz *et al*., 2014). Thus, in the long run, *P. bosniacus* should disperse within the extended range of *D. villosus*, as has been the case in the Rhine–Main–Danube waterways (David *et al*., 2018; Reier *et al*., 2019). This implies, however, that *P. bosniacus* has not performed any intermediate host captures in the novel area yet.

### Host capture

The most interesting finding of this study is the presence of *P. tereticollis* in *G. tigrinus*. In North America, this brackish water amphipod serves as the intermediate host of another *Pomphorhynchus* species, *P. rocci* (Johnson and Harkema, 1971). However, our molecular and morphological data confirmed that the cystacanths harboured by *G. tigrinus* belong indeed to *P. tereticollis* and not to *P. rocci*. While the latter is described as having 15–18 hooks in each longitudinal row, the redescription of *P. tereticollis* postulates 8–12 hooks per row, fitting our observations (Cordonnier and Ward, 1967; Špakulová *et al*., 2011).

Considering a significant drop in attention to the parasite communities of the Weser system in the new millennium, it can be assumed that the host capture of *G. tigrinus* by *P. tereticollis* took place rather recently, as the characteristically orange larvae would not have remained undiscovered for several decades. On the contrary, no parasitological studies on *G. tigrinus* in the Weser system were published between 1987 and till date. Therefore, the host capture possibly happened after a period of 40 or more years of the 65 years of co-habitation.

The capture of the non-native host by *P. tereticollis* might have happened prior to the arrival of Ponto-Caspian invasive species in 1998, when the donor, probably *G. pulex*, as well as the target host *G. tigrinus*, temporarily coexisted in the Weser (Grabow *et al*., 1998). As shown here, the *G. pulex* population in the Fulda is parasitized by *P. laevis*, reducing its potential as the donor host for *P. tereticollis*. It is yet unclear if the host capture is a product of water quality management that resulted in a decrease of salinity (Bäthe and Coring, 2011). In contrast, no evidence of a *P. tereticollis* presence in the upper Werra was discovered; therefore, a return from the unpolluted river sections seems unlikely, though not impossible. Genetic comparison also suggests the cystacanths are closer related to specimen from inland waters rather than of a Baltic Sea origin (Špakulová *et al*., 2011; Perrot-Minnot *et al*., 2018).

### Host specificity

As information on the intermediate host specificity of *Pomphorhynchus* species is scarce, the data presented here add new insights to the puzzling taxonomy of this genus. Contrasts in host patterns should be considered when dealing with parasite taxonomy, as demonstrated by *P.* cf. *minutus.* In Germany and France, it was shown to comprise 3 cryptic species, each revealing a high degree of intermediate host specificity (Zittel *et al*., 2018). *Polymorphus* type 1 only occurred in *Gammarus fossarum* Koch, 1836 and type 2 in *Echinogammarus ischnus* (Stebbing, 1899) and *Echinogammarus berilloni* (Catta, 1878), whereas type 3 (PspT3) was restricted to *G. pulex* and *G. roeselii*. Morphological comparison also revealed the latter type to be more different than the other 2, suggesting it might be a non-indigenous form that was introduced with *G. roeselii* and captured *G. pulex* as a novel host (Grabner *et al*., 2020). Nevertheless, certain variabilities in the morphological features of the proboscis seem inherent to acanthocephalans (Jirsa *et al*., 2022). Considering its potential for host adaptation, PspT3 is the most likely candidate to assign the specimen obtained in this study from *G. tigrinus*. *Gammarus roeselii* can be found in the unpolluted upstream regions of the Werra and both *G. roeselii* and *G. pulex* were found in the Fulda, where also a single *G. pulex* was infected with *P.* cf. *minutus*. Comparison of the single sequence obtained from a Werra specimen against the NCBI database also confirms the assignment to PspT3. As of now, however, due to the lack of more samples, further perspective for the record of *P.* cf. *minutus* in *G. tigrinus* cannot be extrapolated. To our best knowledge, this host–parasite combination has never been mentioned before.

## Conclusion

To summarize, the host–parasite associations between acanthocephalans and their amphipod intermediate hosts described here highlight the Weser river system's bio- and especially xenodiversity. Following the jump invasion to an area already colonized by its natural intermediate host *G. tigrinus*, *P. ambiguus* did not perform any host captures. In contrast, *G. tigrinus* was captured as a novel host by the European *P. tereticollis* within approximately 65 years after the introduction of the American amphipod into the Werra. In contrast, *P. bosniacus* colonized the Weser together with its invasive host *D. villosus* and did not show any host captures within the last 2 decades after the arrival of *D. villosus* in the habitat. Our study exemplifies that in the present times of ecological globalization, communities of hosts and parasites and their associations can be subjected to rapid change and evolution within a few decades.

## Data Availability

Selected sequences obtained for this study were deposited in NCBI GenBank under the accession numbers ON649686–ON649697 and OQ360657. Additional data not included in this article may be requested from the corresponding author.

## Supplementary material

For supplementary material accompanying this paper visit https://doi.org/10.1017/S0031182023000124. click here to view supplementary material

## References

[ref1] Amin OM, Abdullah SMA and Mhaisen FT (2003) Description of *Pomphorhynchus spindletruncatus* n. sp. (Acanthocephala: Pomphorhynchidae) from freshwater fishes in northern Iraq, with the erection of a new pomphorhynchid genus, *Pyriproboscis* n. g., and keys to genera of the Pomphorhynchida. Systematic Parasitology 54, 229–235. 12652074 10.1023/a:1022654921523

[ref2] Andreou D, Antognazza CM, Williams CF, Bradley H, Reading AJ, Hardouin EA, Stewart JR, Sheath DJ, Galligar A, Johnson E and Britton JR (2020) Vicariance in a generalist fish parasite driven by climate and salinity tolerance of hosts. Parasitology 147, 1658–1664. 32907651 10.1017/S0031182020001663 PMC10317739

[ref3] Bandelt HJ, Forster P and Röhl A (1999) Median-joining networks for inferring intraspecific phylogenies. Molecular Biology and Evolution 16, 37–48. 10331250 10.1093/oxfordjournals.molbev.a026036

[ref4] Bäthe J and Coring E (2011) Biological effects of anthropogenic salt-load on the aquatic fauna: a synthesis of 17 years of biological survey on the rivers Werra and Weser. Limnologica 41, 125–133.

[ref5] Boets P, Lock K, Messiaen M and Goethals PLM (2010) Combining data-driven methods and lab studies to analyse the ecology of *Dikerogammarus villosus*. Ecological Informatics 5, 133–139.

[ref6] Braukmann U and Böhme D (2011) Salt pollution of the middle and lower sections of the river Werra (Germany) and its impact on benthic macroinvertebrates. Limnologica 41, 113–124.

[ref7] Bullock WL and Samuel G (1975) *Paratenuisentis* gen. n. for *Tanaorhamphus ambiguus* Van Cleave 1921 (Acanthocephala), with a reconsideration of the Tenuisentidae. The Journal of Parasitology 61, 105–109. 1117351

[ref8] Bulnheim HP (1976) *Gammarus tigrinus*, ein neues Faunenelement der Ostseeforde Schlei. Schriften des Naturwissenschaftlichen Vereins für Schleswig-Holstein 46, 79–84.

[ref9] Cordonnier LM and Ward HL (1967) *Pomphorhynchus rocci* sp. n. (Acanthocephala) from the rock bass, *Roccus saxatilis*. The Journal of Parasitology 53, 1295–1297. 5624886

[ref10] Daunys D and Zettler ML (2006) Invasion of the North American amphipod (*Gammarus tigrinus* Sexton, 1939) into the Cronian Lagoon, South-Eastern Baltic Sea. Acta Zoologica Lituanica 16, 20–26.

[ref11] David GM, Staentzel C, Schlumberger O, Perrot-Minnot M-J, Beisel JN and Hardion L (2018) A minimalist macroparasite diversity in the round goby of the Upper Rhine reduced to an exotic acanthocephalan lineage. Parasitology 145, 1020–1026. 29229008 10.1017/S0031182017002177

[ref12] Dezfuli BS and Giari L (1999) Amphipod intermediate host of *Polymorphus minutus*, parasite of water birds, with notes on ultrastructure of host–parasite interface. Folia Parasitologica 46, 117–122.

[ref13] Dzido J, Rolbiecki L, Izdebska JN and Bednarek R (2020) Checklist of the parasites of European eel *Anguilla anguilla* (Linnaeus, 1758) (Anguillidae) in Poland. Biodiversity Data Journal 8, e52346. 32581635 10.3897/BDJ.8.e52346 PMC7303223

[ref14] Emde S, Rueckert S, Palm HW and Klimpel S (2012) Invasive Ponto-Caspian amphipods and fish increase the distribution range of the acanthocephalan *Pomphorhynchus tereticollis* in the river Rhine. PLoS ONE 7, e53218. 23300895 10.1371/journal.pone.0053218 PMC3534018

[ref15] Gallardo B, Errea MP and Aldridge DC (2012) Application of bioclimatic models coupled with network analysis for risk assessment of the killer shrimp, *Dikerogammarus villosus*, in Great Britain. Biological Invasions 14, 1265–1278.

[ref16] García-Varela M, Mendoza-Garfias B, Choudhury A and Pérez-Ponce de León G (2017) Morphological and molecular data for a new species of *Pomphorhynchus* Monticelli, 1905 (Acanthocephala: Pomphorhynchidae) in the Mexican redhorse *Moxostoma austrinum* Bean (Cypriniformes: Catostomidae) in central Mexico. Systematic Parasitology 94, 989–1006. 29027090 10.1007/s11230-017-9756-y

[ref17] Gilbert S and Bullock WL (1981) Life cycle of *Paratenuisentis ambiguus* (Van Cleave, 1921) Bullock and Samuel, 1975 (Acanthocephala: Tenuisentidae). Journal of Parasitology 67, 214–217. 1117351

[ref18] Grabner DS, Doliwa A, Bulantová J, Horák P and Sures B (2020) Morphological comparison of genetically differentiated *Polymorphus* cf. *minutus* types. Parasitology Research 119, 153–163. 31786696 10.1007/s00436-019-06525-1

[ref19] Grabow K, Eggers TO and Martens A (1998) *Dikerogammarus villosus* Sovinsky (Crustacea: Amphipoda) in norddeutschen Kanälen und Flüssen. Lauterbornia 33, 103–107.

[ref20] Hohenadler MAA, Nachev M, Thielen F, Taraschewski H, Grabner DS and Sures B (2018) *Pomphorhynchus laevis*: an invasive species in the river Rhine? Biological Invasions 20, 207–217.

[ref21] Hübner G (2007) Ökologisch-faunistische Fließgewässerbewertung am Beispiel der salzbelasteten unteren Werra und ausgewählter Zuflüsse.

[ref22] Jirsa F, Reier S and Smales LR (2022) Helminths of the mallard *Anas platyrhynchos* Linnaeus, 1758 from Austria, with emphasis on the morphological variability of *Polymorphus minutus* Goeze, 1782. Journal of Helminthology 95, 1–10. 10.1017/S0022149X21000079 33736731

[ref23] Johnson CA and Harkema R (1971) The life history of *Pomphorhynchus rocci* Cordonnier and Ward, 1967 (Acanthocephala) in the striped bass, *Morone saxatilis*. ASB Bulletin 18, 40.

[ref24] Kaldonski N, Perrot-Minnot M-J, Motreuil S and Cézilly F (2008) Infection with acanthocephalans increases the vulnerability of *Gammarus pulex* (Crustacea, Amphipoda) to non-host invertebrate predators. Parasitology 135, 627–632. 18371238 10.1017/S003118200800423X

[ref25] Kiskároly M and Čanković M (1967) *Pomphorhynchus bosniacus* nov. sp. aus Barben *Barbus barbus* (L.) des Save-Gebietes. Zoologischer Anzeiger 182, 69–74.

[ref26] Kornis MS, Mercado-Silva N and vander Zanden MJ (2012) Twenty years of invasion: a review of round goby *Neogobius melanostomus* biology, spread and ecological implications. Journal of Fish Biology 80, 235–285. 22268429 10.1111/j.1095-8649.2011.03157.x

[ref27] Kumar S, Stecher G, Li M, Knyaz C and Tamura K (2018) MEGA X: molecular evolutionary genetics analysis across computing platforms. Molecular Biology and Evolution 35, 1547–1549. 29722887 10.1093/molbev/msy096 PMC5967553

[ref28] Leigh JW and Bryant D (2015) PopART: full-feature software for haplotype network construction. Methods in Ecology and Evolution 6, 1110–1116.

[ref29] Leuven RSEW, van der Velde G, Baijens I, Snijders J, van der Zwart C, Lenders HJR and Bij de Vaate A (2009) The river Rhine: a global highway for dispersal of aquatic invasive species. Biological Invasions 11, 1989–2008.

[ref30] Li L, Chen HX, Amin OM and Yang Y (2017) Morphological variability and molecular characterization of *Pomphorhynchus zhoushanensis* sp. nov. (Acanthocephala: Pomphorhynchidae), with comments on the systematic status of *Pomphorhynchus* Monticelli, 1905. Parasitology International 66, 693–698. 28629829 10.1016/j.parint.2017.05.010

[ref31] MacNeil C (2019) Predatory impacts of the invasive ‘killer shrimp’ *Dikerogammarus villosus* on a resident amphipod and isopod (Crustacea: Malacostraca) are influenced by water quality and habitat type. Hydrobiologia 833, 53–64.

[ref32] Mauer K, Hellmann SL, Groth M, Fröbius AC, Zischler H, Hankeln T and Herlyn H (2020) The genome, transcriptome, and proteome of the fish parasite *Pomphorhynchus laevis* (Acanthocephala). PLoS ONE 15, 1–30. 10.1371/journal.pone.0232973 PMC7310846 32574180

[ref33] Moret Y, Bollache L, Wattier RA and Rigaud T (2007) Is the host or the parasite the most locally adapted in an amphipod–acanthocephalan relationship? A case study in a biological invasion context. International Journal for Parasitology 37, 637–644. 17266962 10.1016/j.ijpara.2006.12.006

[ref34] Nedić Z, Vardić Smrzlić I, Paraš S and Nikolić V (2019) *Pomphorhynchus bosniacus* Kiškarolj & Čanković 1969 (Acanthocephala), intestinal parasite from the Sava river, Bosnia and Herzegovina: new insights on phylogeny, infection dynamics and histopathology. Bulletin of the European Association of Fish Pathologists 39, 93–105.

[ref35] Perrot-Minnot M-J (2004) Larval morphology, genetic divergence, and contrasting levels of host manipulation between forms of *Pomphorhynchus laevis* (Acanthocephala). International Journal for Parasitology 34, 45–54. 14711589 10.1016/j.ijpara.2003.10.005

[ref36] Perrot-Minnot M-J, Špakulová M, Wattier RA, Kotlík P, Düşen S, Aydoğdu A and Tougard C (2018) Contrasting phylogeography of two Western Palaearctic fish parasites despite similar life cycles. Journal of Biogeography 45, 1–15.

[ref37] Platvoet D, Dick JTA, MacNeil C, van Riel MC and van der Velde G (2009) Invader–invader interactions in relation to environmental heterogeneity leads to zonation of two invasive amphipods, *Dikerogammarus villosus* (Sowinsky) and *Gammarus tigrinus* Sexton: amphipod pilot species project (AMPIS) report 6. Biological Invasions 11, 2085–2093.

[ref38] Reier S, Sattmann H, Schwaha T, Harl J, Konecny R and Haring E (2019) An integrative taxonomic approach to reveal the status of the genus *Pomphorhynchus* Monticelli, 1905 (Acanthocephala: Pomphorhynchidae) in Austria. International Journal for Parasitology: Parasites and Wildlife 8, 145–155. 30788212 10.1016/j.ijppaw.2019.01.009 PMC6369135

[ref39] Reisalu G, Kotta J, Herkül K and Kotta I (2016) The invasive amphipod *Gammarus tigrinus* Sexton, 1939 displaces native gammarid amphipods from sheltered macrophyte habitats of the Gulf of Riga. Aquatic Invasions 11, 45–54.

[ref40] Rewicz T, Grabowski M, MacNeil C and Bącela-Spychalska K (2014) The profile of a ‘perfect’ invader – the case of killer shrimp, *Dikerogammarus villosus*. Aquatic Invasions 9, 267–288.

[ref41] Rewicz T, Grabowski M, Tończyk G, Konopacka A and Bącela-Spychalska K (2019) *Gammarus tigrinus* Sexton, 1939 continues its invasion in the Baltic Sea: first record from Bornholm (Denmark). BioInvasions Records 8, 862–870.

[ref42] Rodjuk G and Shelenkova O (2006) Parasite fauna of the European eel (*Anguilla anguilla* L. 1758) from the Russian part of the Vistula Lagoon (Baltic Sea). Wiadomości parazytologiczne 52, 121–125. 17120994

[ref43] Ros AFH, Basen T, Teschner RJ and Brinker A (2020) Morphological and molecular data show no evidence of the proposed replacement of endemic *Pomphorhynchus tereticollis* by invasive *P. laevis* in salmonids in southern Germany. PLoS ONE 15, 1–17. 10.1371/journal.pone.0234116 PMC7297375 32544162

[ref44] Schmitz W (1960) Die Einbürgerung von *Gammarus tigrinus* Sexton auf dem europäischen Kontinent. Archiv für Hydrobiologie 57, 223–225.

[ref45] Schulz CJ and Cañedo-Argüelles M (2019) Lost in translation: the German literature on freshwater salinization. Philosophical Transactions of the Royal Society B: Biological Sciences 374, 20180007. 10.1098/rstb.2018.0007 PMC6283970 30509909

[ref46] Špakulová M, Perrot-Minnot M-J and Neuhaus B (2011) Resurrection of *Pomphorhynchus tereticollis* (Rudolphi, 1809) (Acanthocephala: Pomphorhynchidae) based on new morphological and molecular data. Helminthologia 48, 268–277.

[ref47] Spikkeland I, Olsen JB, Kasbo R, Olsen KM and Nilssen JP (2020) The invasive amphipod *Gammarus tigrinus* Sexton, 1939 conquering the north of Europe using a new pathway: the first recordings from Norway. Fauna norvegica 40, 130–136.

[ref48] Sures B, Knopf K, Würtz J and Hirt J (1999) Richness and diversity of parasite communities in European eels *Anguilla anguilla* of the river Rhine, Germany, with special reference to helminth parasites. Parasitology 119, 323–330. 10503258 10.1017/s0031182099004655

[ref49] Szöcs E, Coring E, Bäthe J and Schäfer RB (2014) Effects of anthropogenic salinization on biological traits and community composition of stream macroinvertebrates. Science of the Total Environment 468–469, 943–949. 10.1016/j.scitotenv.2013.08.058 24080419

[ref50] Taraschewski H (2006) Hosts and parasites as aliens. Journal of Helminthology 80, 99–128. 16768855 10.1079/joh2006364

[ref51] Taraschewski H, Moravec F, Lamah T and Anders K (1987) Distribution and morphology of two helminths recently introduced into European eel populations: *Anguillicola crassus* (Nematoda, Dracunculoidea) and *Paratenuisentis ambiguus* (Acanthocephala, Tenuisentidae). Diseases of Aquatic Organisms 3, 167–176.

[ref52] Tierney PA, Caffrey JM, Vogel S, Matthews SM, Costantini E and Holland CV (2020) Invasive freshwater fish (*Leuciscus leuciscus*) acts as a sink for a parasite of native brown trout *Salmo trutta*. Biological Invasions 22, 2235–2250.

[ref53] Vardić Smrzlić I, Valić D, Kapetanović D, Filipović Marijić V, Gjurčević E and Teskeredžić E (2015) *Pomphorhynchus laevis* (Acanthocephala) from the Sava river basin: new insights into strain formation, mtDNA-like sequences and dynamics of infection. Parasitology International 64, 243–250. 25728305 10.1016/j.parint.2015.02.004

[ref54] Zittel M, Grabner DS, Wlecklick A, Sures B, Leese F, Taraschewski H and Weigand AM (2018) Cryptic species and their utilization of indigenous and non-indigenous intermediate hosts in the acanthocephalan *Polymorphus minutus* sensu lato (Polymorphidae). Parasitology 145, 1421–1429. 29455678 10.1017/S0031182018000173

