# Supplementary figures and images for: Intermediate host patterns of acanthocephalans in the Weser river system: co-invasion vs host capture
Source: Parasitology. 2023 Feb 16;150(5):426–33. doi: 10.1017/S0031182023000124 (PMC10089806; doi:10.1017/S0031182023000124)

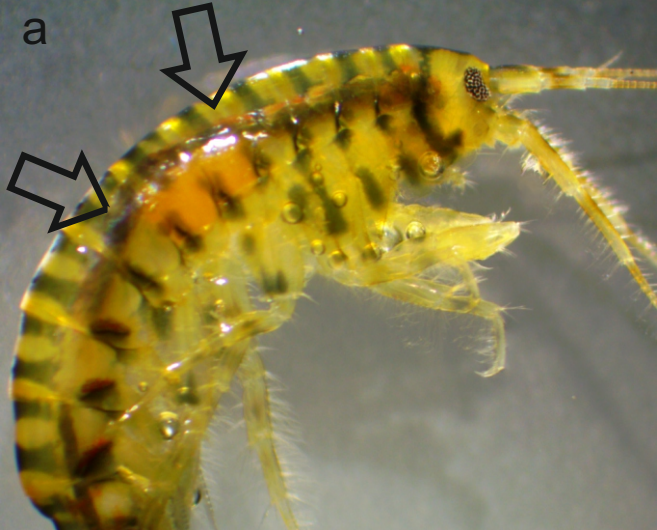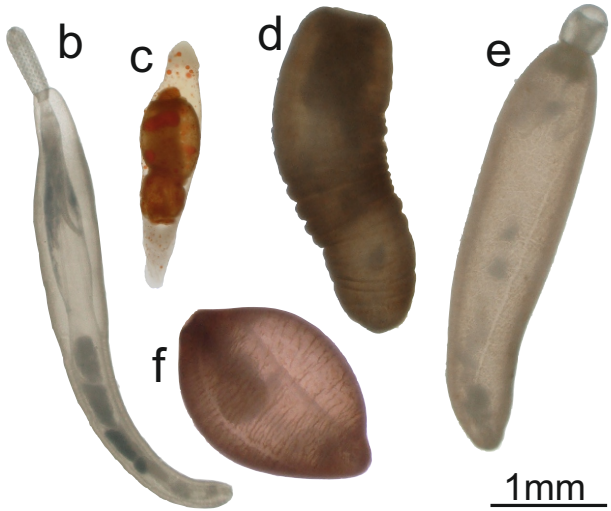

Supplement: Supplementary file 1 [file S0031182023000124sup001.zip › S0031182023000124sup001.pdf]

Fig. S2a MJ-haplotype network of *P. bosniacus*

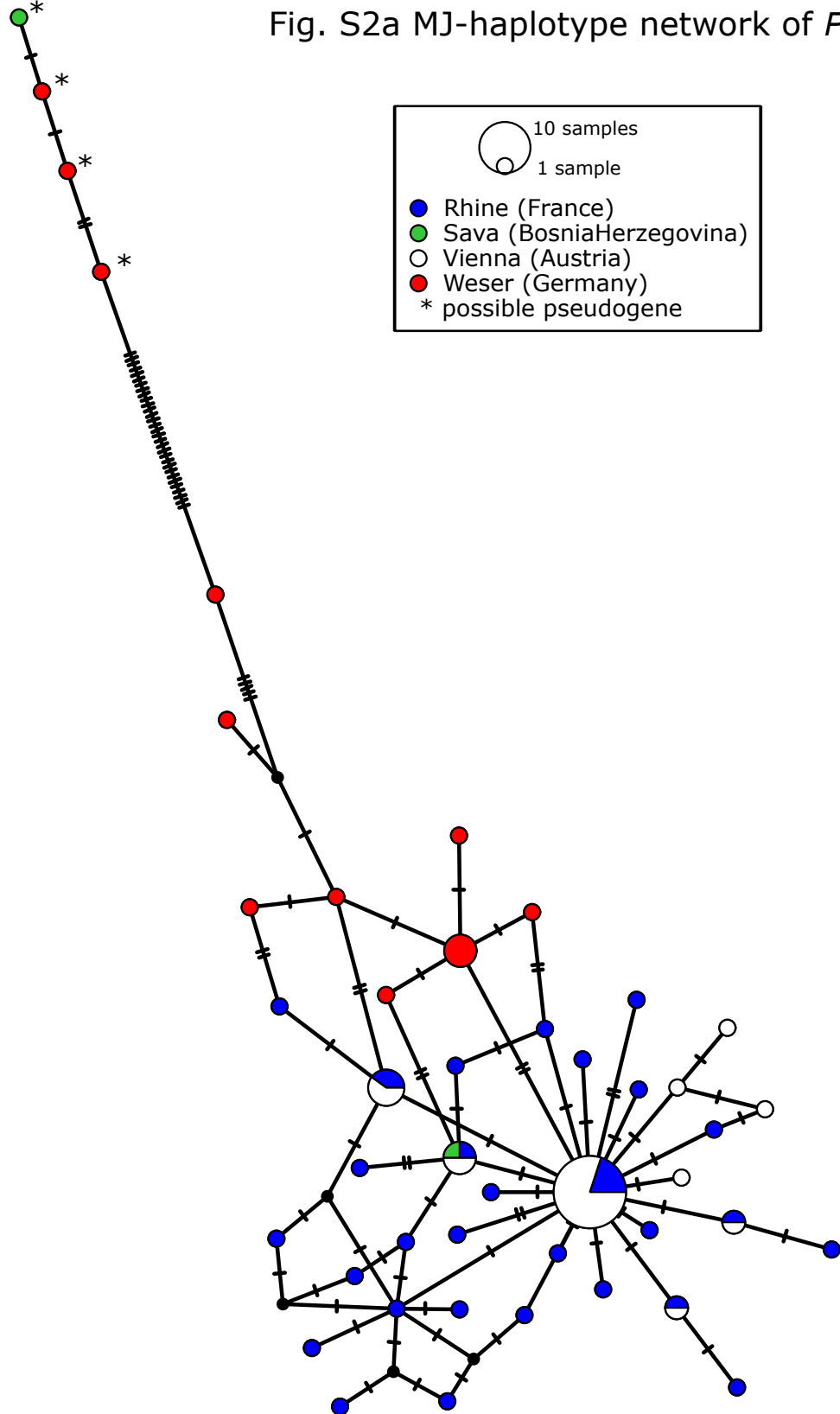

Supplement: Supplementary file 1 [file S0031182023000124sup001.zip › S0031182023000124sup002.pdf]

Fig. S2b MJ-haplotype network of *P. laevis*

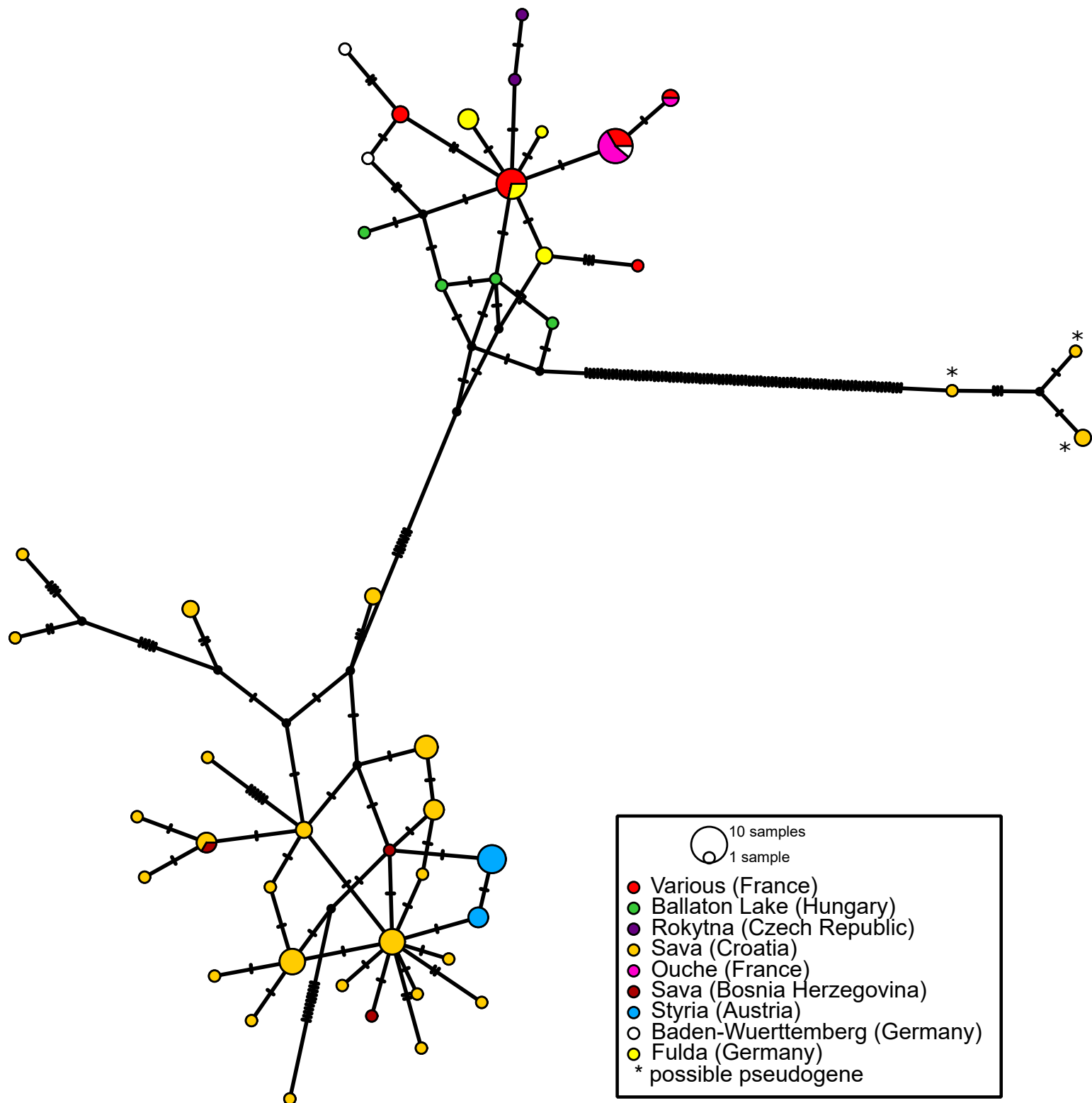

Supplement: Supplementary file 1 [file S0031182023000124sup001.zip › S0031182023000124sup003.pdf]

Fig. S2c MJ-haplotype network of *P. tereticollis*

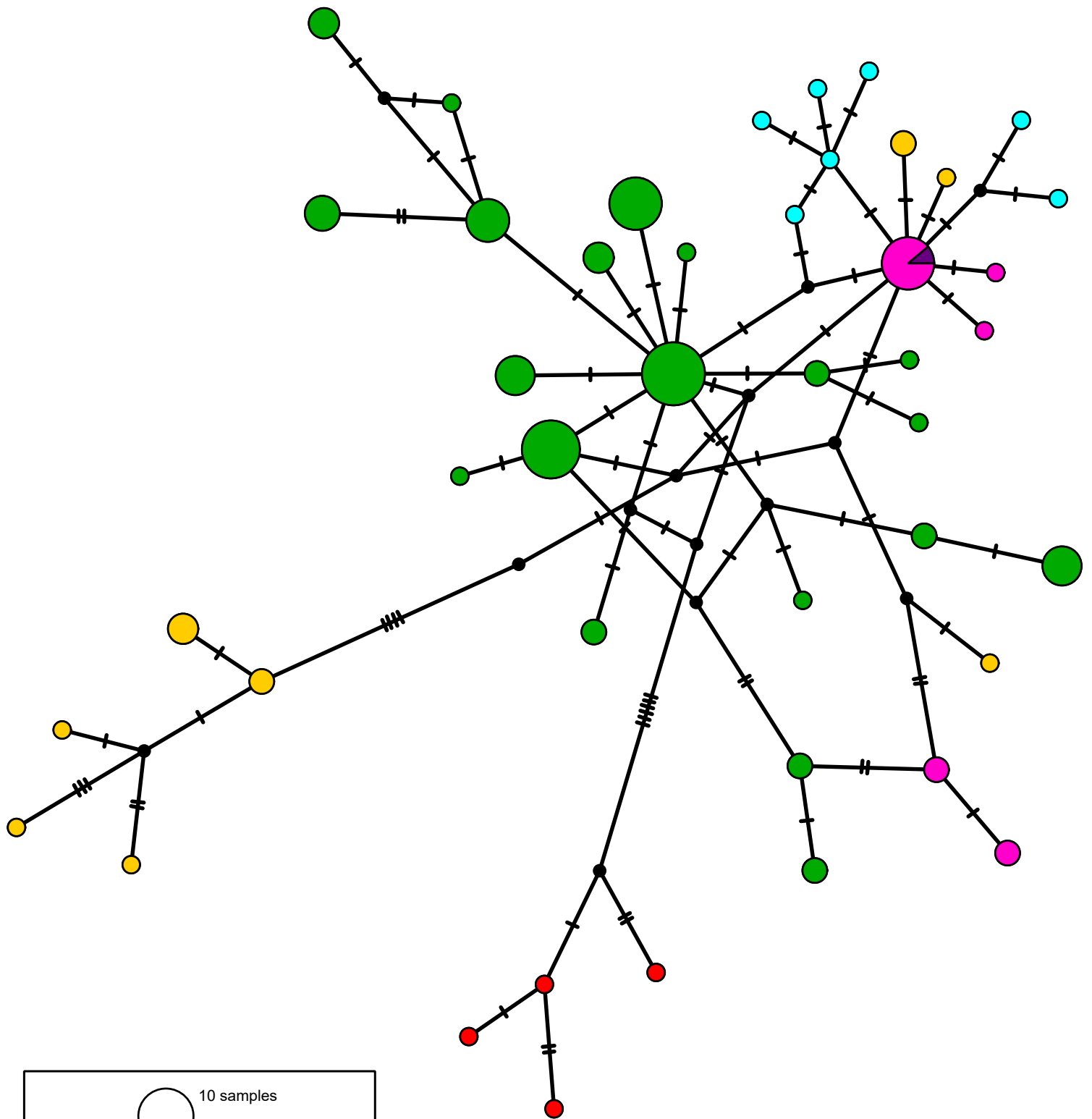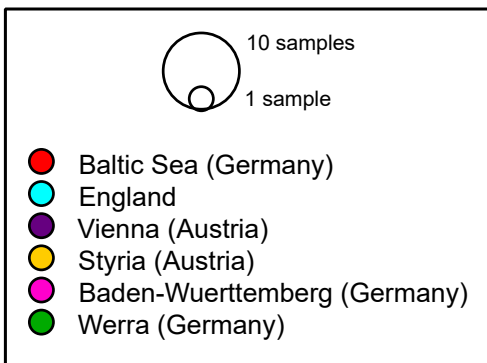

Supplement: Supplementary file 1 [file S0031182023000124sup001.zip › S0031182023000124sup004.pdf]
